# A Practical Treatise on Diseases of the Skin

**Published:** 1891-07

**Authors:** 


A Practical Treatise on Diseases of the Skin. By Henry G. Piffard, M. D., Clinical Professor of Dermatology, University of the City of New York; Surgeon in charge of the New York Dispensary for Diseases of the Skin; Consulting Surgeon to Charity Hospital, etc., etc. Assisted by Robert M. Fuller, M. D. With fifty full-page original plates, and thirty-three illustrations in the text. Large quarto, pp. vi.—159. New York: D. Appleton & Company. London: Caxton House, Paternoster Square. 1891. Price, $15.00.

The scope of this excellent work embraces all that belongs to practical dermatology. It is complete and comprehensive, yet so abridged and condensed as to present the essential details of diagnosis and treatment. The merits and intrinsic worth of the treatise under consideration, may be further attested to by the numerous publications of the author so familiar to the profession. He has undertaken to supply a want which shall serve as a guide to the general practitioner, with the fewest technicalities, steering clear of all theoretical and controversial discussions, and entirely ignoring the pathological histology of the skin, which he claims is still in an extremely inchoate state. However, the most notable facts regarding etiology, diagnosis and methods of treatment are concise and clearly stated, commending themselves as highly practical, and indispensable to the busy doctor.

The preliminary remarks are devoted to diagnosis, especial stress being placed upon the importance of the identification of the almost infinite variety of cutaneous affections, in order that their management and treatment may be more successful. The various diseases of the skin are then accurately described; under the heading of each, brief reference is made to etiology, differential diagnosis, and therapeutics.

Another feature of this most superb text-book and atlas is its illustrations, being eighty-three in number, which comprise fifty plates, and thirty-three minor representations. The plates are excellent specimens of photographic copies from the originals. They are all admirably executed, being reproduced from photographs taken from nature by the aid of artificial light, which the author prefers to ordinary daylight. And, in many instances, he has aimed to bring together as many of the varieties of a given disease as possible, with careful and most accurate delineation.

